# Development of Asymmetric Facial Depigmentation in a Patient Treated with Dasatinib with New-Onset Hypovitaminosis D: Case Report and Review of the Literature

**DOI:** 10.1155/2017/9359086

**Published:** 2017-03-23

**Authors:** Kirsten C. Webb, Magdalena Harasimowicz, Monica Janeczek, Jodi Speiser, James Swan, Rebecca Tung

**Affiliations:** ^1^Department of Dermatology, Loyola University Chicago, Chicago, IL, USA; ^2^Stritch School of Medicine, Loyola University Chicago, Chicago, IL, USA; ^3^Department of Pathology, Loyola University Chicago, Chicago, IL, USA

## Abstract

Dasatinib is a second-generation tyrosine kinase inhibitor (TKI) used to treat imatinib-resistant chronic myelogenous leukemia (CML), as well as other Philadelphia chromosome-positive lymphoproliferative disorders. While the most commonly reported cutaneous side effects with this therapy include a morbilliform eruption, skin exfoliation, and skin irritation, pigmentary abnormalities have also been observed, albeit much more rarely. We present the case of a 72-year-old South Asian male with CML who presented with new-onset hypopigmentation of his face and scalp three years after a dose increase of dasatinib therapy, in the setting of newly discovered borderline hypovitaminosis D. Dasatinib and the other TKIs are believed to induce dyschromias via modulation of the c-kit receptor and its associated signaling pathway, which is involved in melanocyte survival, proliferation, and migration.

## 1. Introduction

Dasatinib is a second-generation tyrosine kinase inhibitor (TKI) used to treat imatinib-resistant chronic myelogenous leukemia (CML), other Philadelphia chromosome-positive lymphoproliferative disorders, and certain solid tumors [1]. The most commonly reported cutaneous side effects with this therapy include morbilliform eruptions, skin exfoliation, and skin irritation [2]. While hypopigmentation can affect up to 41% of patients treated with imatinib [3], it is much more rarely reported in patients treated with second-generation TKIs, such as dasatinib. We present a case of dasatinib-associated dyschromia in the setting of newfound borderline hypovitaminosis D and review those cases in the literature.

## 2. Case

**Figure 1:**
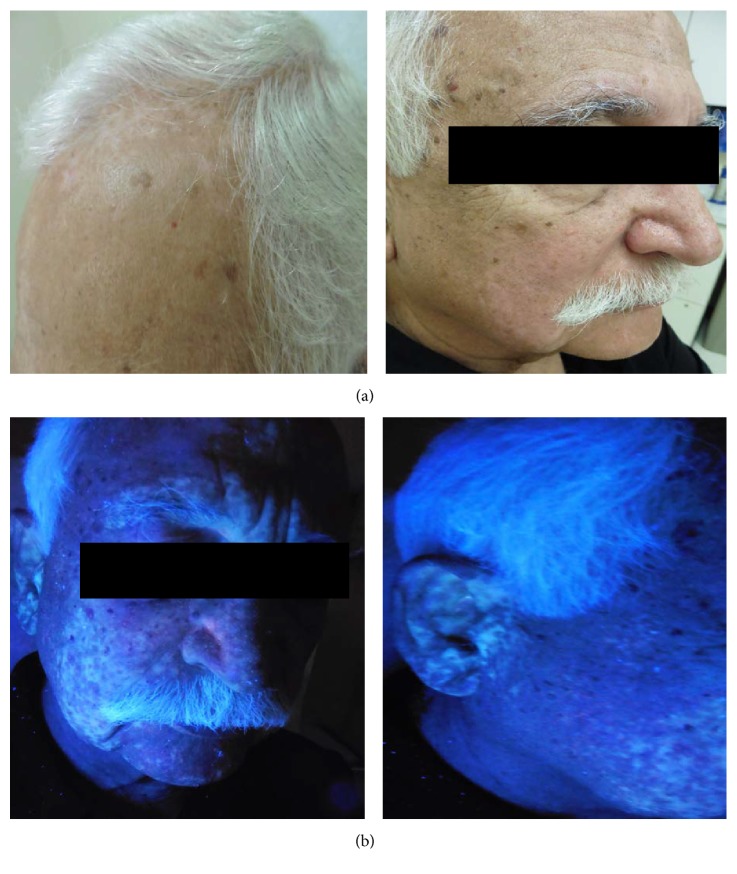
Hypopigmented and depigmented areas on the (a) face, scalp, and (b) ears, highlighted on Wood's lamp examination.

A 72-year-old South Asian male presented with new-onset hypopigmented patches on his frontal scalp, cheeks, and forehead of four weeks' duration. He had a past medical history significant for imatinib-resistant CML, currently being treated with dasatinib. There was no personal or family history of autoimmune diseases, pigmentary disorders, or melanoma. Of note, the patient had been diagnosed with CML 13 years priorly and was successfully treated with imatinib 400 mg po daily for 10 years. However, over nine months' time, the patient's quantitative Bcr-Abl fusion transcript product serum level subsequently rose from an undetectable amount (zero) to 1.443. This prompted a repeat bone marrow biopsy, which revealed a novel heterozygous point mutation, c.1003G>A, within the Abl kinase domain. At this time, the patient underwent a therapeutic switch from imatinib to dasatinib. The patient had noted diffuse skin lightening of the head and neck while treated with imatinib, which subsequently resolved in its entirety after he was transitioned to dasatinib. He was initiated on dasatinib therapy at a dose of 50 mg daily for the first 10 weeks, and then his dose was increased to 100 mg daily. Three years after this dose escalation, the patient presented to dermatology clinic with new-onset hypopigmented and depigmented macules and patches of varying sizes on his superior forehead, bilateral melolabial cheeks, and chin (Figure 1(a)). Additionally, confetti-like depigmentation was present on the bilateral ears. These achromic lesions were more fully appreciated on Wood's lamp examination (Figure 1(b)). All of the patient's scalp hair and the majority of the facial hair were depigmented. Laboratory workup revealed a low normal serum vitamin D level. Shave biopsy of a representative lesion on the frontal scalp revealed a decreased melanocyte number on MART-1 staining, and PAS staining was negative for fungal organisms. The patient's hypopigmented lesions were attributed to his dasatinib therapy.

## 3. Discussion

Dyschromias are rarely reported in patients treated with dasatinib and other second-generation TKIs. In reported cases of dasatinib-associated dyschromias (Table 1), pigmentary changes began four weeks to six months following treatment initiation and appear to have a predilection for the head and neck [1, 4–7]. Our patient's cutaneous pigmentary changes were noted approximately three years (37.5 months) after treatment initiation, which is the longest time to onset reported as of yet. His hair depigmentation predated his treatment with any TKI. Dyspigmentation appears to be dose-dependent, and repigmentation is achievable with cessation of dasatinib therapy. In reported cases, repigmentation began as early as four to eight weeks following withdrawal of therapy [1, 7]. Unfortunately, while effective, this is not a practical treatment approach in most cases, including our own patient's case, as patients' underlying malignancies often necessitate continued treatment with the offending TKI.

Dasatinib targets multiple mutant forms of the Bcr-Abl protein, as well as the SRC family of kinases, c-kit, and Platelet-Derived Growth Factor Receptor *β* (PDGFR-*β*) tyrosine kinases [8]. Dasatinib and other TKIs are thought to induce dyschromias via inhibition of the protooncogene, c-kit. C-kit encodes a class III transmembrane tyrosine kinase receptor found on the surface of melanocytes and hematopoietic stem cells [1, 9–11]; its ligand is stem cell factor (SCF). Upon binding with SCF, c-kit undergoes dimerization and autophosphorylation, which activates downstream pathways involved in melanocyte proliferation, migration, and survival [9–11].

There is ample support for c-kit's role in melanocyte survival and functioning. C-kit's role in melanocyte migration is highlighted by the clinical disorder, piebaldism [12]. Affected patients have c-kit mutations, resulting in failed proliferation or migration of melanoblasts from the neural crest during embryonic development to their appropriate destination in the skin [12]. This results in the clinical findings of white skin patches and white hair, most commonly on the forehead, trunk, and extremities [13]. C-kit's role in melanocyte proliferation is demonstrated in a study in which human skin xenografts treated with KIT inhibitory antibodies resulted in a decrease in melanocyte number [9]. C-kit also appears to play a role in melanocyte functioning. Skin samples from mice treated with sunitinib, a TKI which inhibits c-kit, showed no change in the number of KIT-positive melanocytes; however, these mice exhibited dose-dependent and reversible hair depigmentation [14]. Clearly, c-kit plays an integral role in melanocyte biology. Thus, it is not surprising that interference with this pathway results in the clinical pigmentary anomalies observed in patients treated with TKIs.

While hypopigmentation and depigmentation are the most commonly observed pigmentary anomalies with TKIs, hyperpigmentation has also been reported. In one series, 3.6% of patients treated with imatinib experienced hyperpigmentation [3]. Moreover, one patient treated with dasatinib initially experienced widespread hypopigmentation. Upon withdrawal of dasatinib, she experienced transient hyperpigmentation before her baseline pigment was restored [1]. These latter observations suggest that, rather than inducing true absolute inhibition of c-kit, TKIs may instead act as c-kit modulators, resulting in a spectrum of possible pigmentary abnormalities. Interestingly, the ability of TKIs to inhibit c-kit activity and signaling may depend on the conformation of c-kit ligand (SCF) present in the tissues, namely, whether SCF is spliced into a membrane-bound or soluble form. Indeed, a recent study showed that membrane-bound c-kit ligand was capable of inducing c-kit mediated signaling independent of kinase functioning and rendered membrane-bound SCF/c-kit receptors insensitive to imatinib. This was found in contrast to their soluble SCF/c-kit receptor counterparts, which were sensitive to this therapy [15].

The reason dyschromias are much more frequently observed with first-generation TKIs compared to second-generation TKIs is not fully understood. There also appears to be a disparity between members of the same TKI generation in terms of their likelihood of inducing pigmentary anomalies. This is demonstrated in a report by Fujimi et al., in which a CML patient experienced reversible dasatinib-induced skin and hair depigmentation and then did not experience any depigmentation when subsequently treated with bosutinib [7]. Bosutinib is a tyrosine kinase inhibitor which targets multiple Bcr-Abl mutant forms but, in contrast to dasatinib and other second-generation TKIs, has little to no affinity for the c-kit and PDGF receptors [16–18]. Variations in receptor affinities afford a possible explanation for the differing frequencies of observed dyschromias between different TKI generations and between individual members of each TKI generation. On a similar note, variations in patients' receptor sequencing and structure may play an important role in determining which patients ultimately develop pigmentary anomalies when treated with TKI therapies. Indeed, the necessity of possessing a certain genetic predisposition and/or having certain environmental exposures could explain why only certain patients are afflicted with these side effects and may also explain the observed differences in time to onset of depigmentation and doses of TKIs required to elicit pigmentary anomalies.

Another important consideration in this case is the role of vitamin D in cutaneous disorders of hypopigmentation. Its various biologic properties have different implications depending upon the dermatologic condition in question. For example, its immunomodulatory properties, including inhibition of the inflammatory and proapoptotic cytokines, IL-6, IL-8, TNF-*α*, and TNF-*γ* [19], inhibition of antigen presentation [20, 21], and its observed depletion (low serum levels) in patients with various autoimmune diseases [22], sparked investigations into its potential role in vitiligo pathogenesis. Although a causative role for vitamin D deficiency in vitiligo has not yet been established [23], topical and systemic vitamin D formulations are often employed in vitiligo treatment given its known immunomodulatory effects. More relevant to our own patient's case are vitamin D's effects on melanocyte biology and survival. Vitamin D plays a role in melanocyte differentiation, maturation, proliferation, migration, and melanogenesis [24]. Importantly, vitamin D has also been shown to have protective effects against melanocyte apoptosis [25, 26]. It exerts these effects via interaction with the nuclear vitamin D receptor (VDR) on melanocytes [27–29]. Our patient experienced only minimal improvement with administration of topical and systemic vitamin D supplementation, suggesting that dasatinib therapy was the main driver of his pigment loss. However, given vitamin D's recognized melanocytic effects as discussed above, it is possible that his borderline hypovitaminosis D led to more pronounced depigmentation at presentation than might have been observed if he had higher baseline serum vitamin D levels.

Since our patient's underlying CML necessitated continued treatment with dasatinib and he declined procedural treatments, we treated his dyschromia topically with mometasone 0.1% and calcipotriene 0.005% creams. We also initiated over-the-counter therapy with oral vitamin D3 (2000 IU) supplementation. These interventions produced modest improvement in his skin findings. Interestingly, the patient's reported remote past history of diffuse skin lightening was probably attributable to his prior treatment with imatinib. Although he never sought treatment at that time, it fully resolved with therapy cessation, as expected. We present this case to highlight a rare cutaneous side effect of a medication that is being utilized with increasing frequency for treatment-resistant hematological malignancies and certain solid tumors. We encourage clinicians to consider this cutaneous side effect in the differential diagnosis of vitiligo, postinflammatory hypopigmentation, and pityriasis versicolor in patients undergoing treatment with dasatinib. Additionally, continued research exploring how the SCF/c-kit pathway and tyrosine kinase receptors impact melanocyte biogenesis and survival could afford further insight into management of patients suffering from vitiligo and other pigmentary disorders.

## Figures and Tables

**Table 1:** Summary of cases reporting dasatinib-induced hypopigmentation.

Age (years)	Malignancy	Dasatinib dose (mg)	Time to onset of depigmentation (weeks)	Time to repigmentation (weeks)	*Ref*
52	Metastatic hemangiopericytoma	70 (twice daily)	8	4–6	*[1]*
29	Chronic myelogenous leukemia	70 (once daily)	6–8	N/A	*[4]*
16	Acute lymphoblastic leukemia	100 (twice daily)	4	N/A	*[5]*
27	Chronic myelogenous leukemia	100 (once daily)	24	N/A	*[6]*
56	Chronic myelogenous leukemia	70 (once daily)	8	8	*[7] *

## References

[1] Boudadi K., Chugh R. (2014). Diffuse hypopigmentation followed by hyperpigmentation in an African American woman with hemangiopericytoma treated with dasatinib. *Journal of Clinical and Diagnostic Research*.

[2] Amitay-Laish I., Stemmer S. M., Lacouture M. E. (2011). Adverse cutaneous reactions secondary to tyrosine kinase inhibitors including imatinib mesylate, nilotinib, and dasatinib. *Dermatologic Therapy*.

[3] Arora B., Kumar L., Sharma A., Wadhwa J., Kochupillai V. (2004). Pigmentary changes in chronic myeloid leukemia patients treated with imatinib mesylate. *Annals of Oncology*.

[4] Sun A., Akin R. S., Cobos E., Smith J. (2009). Hair depigmentation during chemotherapy with dasatinib, a dual Bcr-Abl/Src family tyrosine kinase inhibitor. *Journal of Drugs in Dermatology*.

[5] Brazzelli V., Grasso V., Barbaccia V. (2012). Hair depigmentation and vitiligo-like lesions in a leukaemic paediatric patient during chemotherapy with dasatinib. *Acta Dermato-Venereologica*.

[6] Samimi S., Chu E., Seykora J. (2013). Dasatinib-induced leukotrichia in a patient with chronic myelogenous leukemia. *JAMA Dermatology*.

[7] Fujimi A., Ibata S., Kanisawa Y. (2016). Reversible skin and hair depigmentation during chemotherapy with dasatinib for chronic myeloid leukemia. *Journal of Dermatology*.

[8] Shayani S. (2010). Dasatinib, a multikinase inhibitor: therapy, safety, and appropriate management of adverse events. *Therapeutic Drug Monitoring*.

[9] Grichnik J. M., Burch J. A., Burchette J., Shea C. R. (1998). The SCF/KIT pathway plays a critical role in the control of normal human melanocyte homeostasis. *Journal of Investigative Dermatology*.

[10] Wehrle-Haller B. (2003). The role of Kit-ligand in melanocyte development and epidermal homeostasis. *Pigment Cell Research*.

[11] Galanis A., Levis M. (2015). Inhibition of c-kit by tyrosine kinase inhibitors. *Haematologica*.

[12] Giebel L. B., Spritz R. A. (1991). Mutation of the KIT (mast/stem cell growth factor receptor) protooncogene in human piebaldism. *Proceedings of the National Academy of Sciences of the United States of America*.

[13] Ezoe K., Holmes S. A., Ho L. (1995). Novel mutations and deletions of the KIT (steel factor receptor) gene in human piebaldism. *American Journal of Human Genetics*.

[14] Moss K. G., Toner G. C., Cherrington J. M., Mendel D. B., Laird A. D. (2003). Hair depigmentation is a biological readout for pharmacological inhibition of KIT in mice and humans. *Journal of Pharmacology and Experimental Therapeutics*.

[15] Tabone-Eglinger S., Calderin-Sollet Z., Pinon P. (2014). Niche anchorage and signaling through membrane-bound Kit-ligand/c-kit receptor are kinase independent and imatinib insensitive. *The FASEB Journal*.

[16] Puttini M., Coluccia A. M. L., Boschelli F. (2006). In vitro and in vivo activity of SKI-606, a novel Src-Abl inhibitor, against imatinib-resistant Bcr-Abl+ neoplastic cells. *Cancer Research*.

[17] Quintás-Cardama A., Kantarjian H., Cortes J. (2007). Flying under the radar: the new wave of BCR-ABL inhibitors. *Nature Reviews Drug Discovery*.

[18] Remsing Rix L. L., Rix U., Colinge J. (2009). Global target profile of the kinase inhibitor bosutinib in primary chronic myeloid leukemia cells. *Leukemia*.

[19] Koizumi H., Kaplan A., Shimizu T., Ohkawara A. (1997). 1,25-Dihydroxyvitamin D3 and a new analogue, 22-oxacalcitriol, modulate proliferation and interleukin-8 secretion of normal human keratinocytes. *Journal of Dermatological Science*.

[20] Penna G., Adorini L. (2000). 1*α*,25-Dihydroxyvitamin D3 inhibits differentiation, maturation, activation, and survival of dendritic cells leading to impaired alloreactive T cell activation. *The Journal of Immunology*.

[21] Griffin M. D., Lutz W., Phan V. A., Bachman L. A., McKean D. J., Kumar R. (2001). Dendritic cell modulation by 1*α*,25 dihydroxyvitamin D3 and its analogs: a vitamin D receptor-dependent pathway that promotes a persistent state of immaturity in vitro and in vivo. *Proceedings of the National Academy of Sciences of the United States of America*.

[22] Hewison M. (2012). An update on vitamin D and human immunity. *Clinical Endocrinology*.

[23] Karagün E., Ergin C., Baysak S., Erden G., Aktaş H., Ekiz Ö. (2016). The role of serum vitamin D levels in vitiligo. *Advances in Dermatology and Allergology*.

[24] Doss R., El-Rifaie A.-A., Gohary Y., Rashed L. (2015). Vitamin D receptor expression in vitiligo. *Indian Journal of Dermatology*.

[25] Mason R. S., Holliday C. J. (2000). 1, 25-Dihydroxyvitamin D contributes to photoprotection in skin cells. *Vitamin D Endocrine System: Structural, Biological, Genetic and Clinical Aspects*.

[26] Sauer B., Ruwisch L., Kleuser B. (2003). Antiapoptotic action of 1*α*,25-dihydroxyvitamin D3 in primary human melanocytes. *Melanoma Research*.

[27] Tomita Y., Torinuki W., Tagami H. (1988). Stimulation of human melanocytes by vitamin D3 possibly mediates skin pigmentation after sun exposure. *Journal of Investigative Dermatology*.

[28] Watabe H., Soma Y., Kawa Y. (2002). Differentiation of murine melanocyte precursors induced by 1,25-dihydroxyvitamin D3 is associated with the stimulation of endothelin B receptor expression. *Journal of Investigative Dermatology*.

[29] AlGhamdi K., Kumar A., Moussa N. (2013). The role of vitamin D in melanogenesis with an emphasis on vitiligo. *Indian Journal of Dermatology, Venereology and Leprology*.

